# Refractory Fungal Keratitis Caused by Acrophialophora fusispora: A Case Report and Literature Review

**DOI:** 10.7759/cureus.43181

**Published:** 2023-08-09

**Authors:** Atsuhiko Fukuto, Tai-ichiro Chikama, Shiho Ota, Takashi Yaguchi, Yoshiaki Kiuchi

**Affiliations:** 1 Department of Ophthalmology and Visual Sciences, Graduate School of Biomedical and Health Sciences, Hiroshima University, Hiroshima, JPN; 2 Section of Clinical Laboratory, Division of Clinical Support, Hiroshima University Hospital, Hiroshima, JPN; 3 Division of Laboratory Medicine, Hiroshima University Hospital, Hiroshima, JPN; 4 Medical Mycology Research Center, Chiba University, Chiba, JPN

**Keywords:** infectious keratitis, therapeutic penetrating keratoplasty, fungal keratitis, voriconazole, acrophialophora

## Abstract

*Acrophialophora fusispora* is a filamentous fungus that is found in soil and rarely infects humans. We herein report the first case of fungal keratitis caused by *A. fusispora* in Japan and present a review of the literature on human infections with *Acrophialophora* species. A 62-year-old Japanese male on immunosuppressive therapy developed fungal keratitis after the removal of a corneal foreign body from his left eye. Voriconazole eye drops and systemic therapy for post-traumatic fungal keratitis did not resolve the infection, and the patient required a therapeutic corneal transplant. The isolate was identified as *A. fusispora* based on the nucleotide sequence of the internal transcribed spacer region. In a drug susceptibility test, the minimum inhibitory concentration of voriconazole was 0.5 μg/mL. Based on this case and previous cases from the literature review, fungal keratitis caused by *A. fusispora* is often refractory.

## Introduction

Fungal keratitis is a severe ocular infection that can result in visual impairment or even blindness if left untreated. Among the various fungal species that can cause keratitis, *Acrophialophora fusispora* is an emerging pathogen that has recently been implicated in several cases of fungal keratitis [1-3]. *Acrophialophora fusispora* is a dematiaceous fungus commonly found in soil and decaying vegetation [4]. Although not a well-known pathogen, *A. fusispora* has been increasingly reported to cause infections in immunocompromised individuals, particularly those with hematologic malignancies, solid organ transplants, and HIV infection [3,5-7]. However, there are limited reports of *A. fusispora*-associated keratitis in the literature. This report presents a case of post-traumatic infectious keratitis caused by *A. fusispora* with the aim of raising awareness of this emerging fungal pathogen as a possible cause of keratitis.

## Case presentation

**Figure 1:**
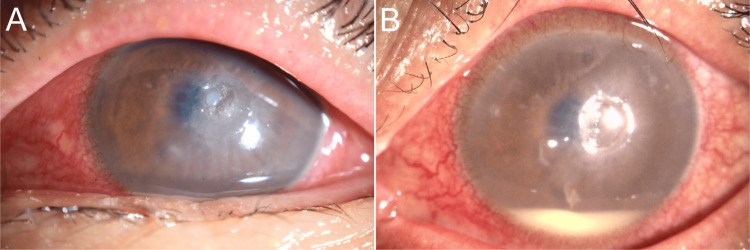
Slit-lamp microscopic image of the left eye. (A) At the first visit, conjunctival hyperemia was prominent, and an infiltrate with indistinct borders was seen in the center of the cornea. (B) One month after the first visit, corneal infiltrates remained, and a 1.5 mm hypopyon was also present.

A 62-year-old male who lived in a rural area of Okayama Prefecture, Japan, and had not recently traveled abroad visited a local general hospital because a stone had entered his left eye while he was mowing in August 2022. He was taking oral steroids and tacrolimus for the treatment of myasthenia gravis. A stony foreign body was found deep in the center of the left cornea, and minor inflammation was present in the anterior chamber. Corneal foreign body removal and anterior chamber washout were performed. Levofloxacin and betamethasone sodium phosphate eye drops were started; however, the corneal epithelial defect persisted, and a feather-like opacity appeared in the injured area. The patient was diagnosed with suspected fungal keratitis, and 1% voriconazole eye drops six times a day and 1% natamycin ointment four times a day were started on day 18 after the injury. Systemic administration of fluconazole and voriconazole was added, but the clinical findings did not improve. Two months after the injury, the patient was referred to the Department of Ophthalmology at Hiroshima University Hospital. Examination revealed an indistinct border infiltrate with an epithelial defect in the center of the cornea, hypopyon, and endothelial plaque (Figure 1A). We diagnosed suspected keratitis caused by filamentous fungi. We treated the patient with natamycin eye drops, voriconazole eye drops, and systemic voriconazole. However, the deep stromal infiltration and hypopyon remained (Figure 1B). Therefore, a therapeutic corneal transplant was performed in the third month after the injury.

**Figure 2:**
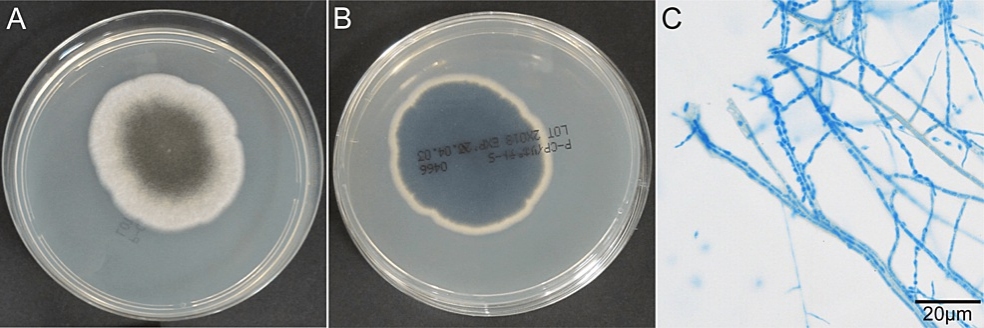
Morphological features of A. fusispora. (A) Front side of the colony after six days on potato dextrose agar at 36°C. (B) Reverse side of the colony. (C) Microscopic morphology of *A. fusispora* stained with cotton blue.

We macroscopically and microscopically observed a pathogenic strain obtained from the excised cornea. The front side of the colony was white to gray in color and fluffy or wooly in texture, and the back side was black (Figure 2A, 2B). The isolate was characterized by pigmented conidiophores arising singly on vegetative hyphae and pale brown and echinulate conidia forming chains (Figure 2C).

**Figure 3:**
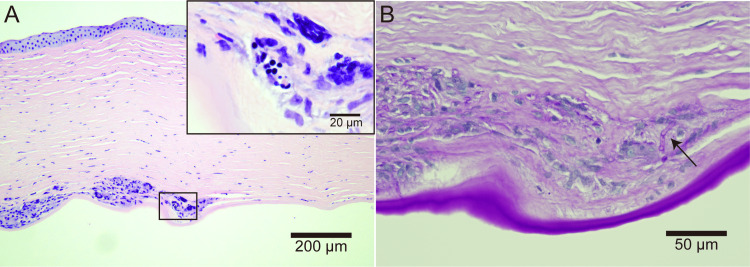
Histopathologic images of the excised cornea. (A) Hematoxylin and eosin stain: neutrophil granulocytes and conidia (inlet) were found in the deep corneal stroma. (B) Periodic acid-Schiff stain: hyphae were found (arrow).

Histopathologic examination of the excised cornea revealed a neutrophilic infiltrate only in the deep layer of the corneal stroma (Figure 3A). Because conidia and hyphae were also present in the same area, we confirmed the diagnosis of fungal keratitis (Figure 3A, 3B).

According to the Basic Local Alignment Search Tool search using the sequence of the ribosomal RNA gene internal transcribed spacer (ITS) domain base, the sequence of this strain showed 100% similarity (548/548) to that of the type strain of *A. nainiana* CBS 100.60 (DDBJ accession number: EM995903). The isolate was thus identified as *A. fusispora* based on morphology and phylogeny. The pathogenic strain in the current case was deposited at the Medical Mycology Research Center at Chiba University, Japan, as IFM 68198 through the National BioResource Project of Japan. The minimum inhibitory concentrations of several antifungal agents to the strain were determined using the broth dilution method according to M38-A2 of the Clinical and Laboratory Standards Institute (Table 1).

**Table 1:** MICs of different antifungal agents. MIC: minimum inhibitory concentration

Antifungal agent	MIC (µg/mL)
Amphotericin B	2
Terbinafine	2
Luliconazole	≤0.008
Itraconazole	0.5
Voriconazole	0.5
Ravuconazole	0.06
Posaconazole	≤0.015

## Discussion

*Acrophialophora fusispora* is a fungus rarely detected in human specimens. To our knowledge, this is the first report of keratitis caused by *A. fusispora* in Japan. The genus *Acrophialophora* was first described by Edward in 1959 as *A. nainiana* [8]. In 2015, Sandoval-Denis et al. performed sequence analysis of the large ribosomal subunit and ITS regions of the nuclear ribosomal DNA and a fragment of the b-tubulin gene (*Tub*), revealing that *Acrophialophora* comprises three species: *A. fusispora*, *A. levis*, and *A. seudatica* [4]. Identification of *Acrophialophora* species by morphological features alone is challenging, and in some cases, they were misdiagnosed as *Scopulariopsis chartarum* or *Scedosporium prolificans* [1,9,10]. Therefore, genetic analysis is essential for the diagnosis of *Acrophialophora* infection. The number of reports in which *Acrophialophora* infection was diagnosed by sequencing of the ITS region has recently been increasing [2,6,7,11,12].

Previous reports of *Acrophialophora* infection in humans are summarized in Table 2. Most reports of *Acrophialophora* infection are from temperate and tropical climates in Asia, southern Europe, and the United States. *Acrophialophora fusispora* and *A. levis* infect the respiratory tract and brain, but only *A. fusispora* has been reported to infect the ocular surface. There are no reports of *A. seudatica* infecting humans. Many patients infected with *Acrophialophora* were immunocompromised, either after organ transplantation or because they had blood diseases. In the present case, the patient was receiving steroids and immunosuppressive drugs to treat myasthenia gravis, and the *A. fusispora* was therefore thought to have been an opportunistic infection. *Acrophialophora* infections are intractable; among previous reports, three of seven patients with lung or brain infections died, and two of three patients with corneal infections required therapeutic corneal transplants.

**Table 2:** Clinical cases of Acrophialophora infection.

Age/gender	Year	Region	Species	Basic disease	Infection site	Antifungal agent	Outcome	Reference
67/male	1997	Spain	A. fusispora	Lung transplantation for pulmonary fibrosis	Lung	Liposomal amphotericin B, itraconazole	Died	[3]
12/female	1998	Sudan	A. fusispora	Acute lymphoblastic leukemia	Brain	Liposomal amphotericin B, itraconazole	Survived	[5]
76/female	2000	United States	A. fusispora	Retained contact lens	Cornea	Not administered	Improved	[1]
33/male	2005	Portugal	A. fusispora	Lung transplantation for post-infective bronchiectasis	Lung	Voriconazole	Improved	[3]
55/female	2005	India	A. fusispora	Not mentioned	Cornea	Fluconazole	Therapeutic keratoplasty	[3]
60/male	2013	Taiwan	A. fusispora	Acquired immunodeficiency syndrome	Brain	Voriconazole	Died	[6]
77/male	2017	Japan	A. fusispora	Neutropenia, hemodialysis, prostate carcinoma	Conjunctiva	Liposomal amphotericin B, itraconazole, voriconazole	Improved	[7]
71/male	2018	China	A. levis	None	Lung	Liposomal amphotericin B, caspofungin	Died	[11]
30/female	2019	India	A. fusispora	Not mentioned	Cornea	Natamycin	Therapeutic keratoplasty	[2]
59/male	2019	India	A. fusispora	Mixed connective tissue disease, chronic pulmonary aspergillosis	Lung	Itraconazole	Improved	[12]
54/female	2019	United States	A. levis	Kidney transplantation	Brain	Voriconazole	Improved	[13]

There are no specific guidelines for treating *Acrophialophora* infection because of its rarity in humans and the scarcity of antifungal susceptibility data. Voriconazole reportedly exhibits higher drug susceptibility to *A. fusispora* than other antifungal agents [4]. The isolate in this study also showed good drug sensitivity to voriconazole. In the present case, despite prolonged systemic and topical administration of voriconazole, the lesion remained in the deep corneal layers and eventually required therapeutic keratoplasty. The reason for this was thought to be that the temperature in the deep layers of the cornea (close to 40°C, the optimum temperature for *A. fusispora*) was higher than that of the superficial layers [4,14]. Luliconazole showed the lowest minimum inhibitory concentration in the drug susceptibility test conducted in this study. Luliconazole is an imidazole antifungal drug widely used as a treatment for ringworm [15]. Luliconazole has been reported to be very effective against the filamentous fungi that cause keratitis [16]. The development of luliconazole eye drops is expected to provide a new treatment option for *Acrophialophora* keratitis.

## Conclusions

This study reports the first case of *Acrophialophora fusispora* keratitis in Japan, emphasizing the crucial role of genetic analysis in diagnosis. Notably, the infection primarily affects immunocompromised individuals, often with severe consequences. The limited efficacy of existing antifungal treatments in this case points to the potential for new therapies, with luliconazole eye drops representing a promising option. Further research is essential to validate these findings and explore new treatment strategies for *Acrophialophora* infections.
